# Emotional Abuse in Childhood and Social Anxiety Symptoms in Adulthood: Examining the Role of Self-Compassion

**DOI:** 10.1007/s40653-026-00870-x

**Published:** 2026-04-06

**Authors:** Zhuoma Ciren, Elsie Ong

**Affiliations:** 1 Department of Psychology & Human Development, IOE – UCL’s Faculty of Education and Society, University College London, London, UK; 2 Faculty of Arts and Social Sciences, Hong Kong Baptist University, Kowloon, Hong Kong SAR China

## Abstract

**Background:**

Childhood emotional abuse has been consistently linked to internalizing difficulties, including social anxiety; however, findings regarding its relative contribution compared to other forms of maltreatment have been mixed. Moreover, little is known about the psychological processes that may account for this association.

**Objective:**

The present study aimed (a) to re-examine the association between childhood emotional abuse and adult social anxiety while accounting for co-occurring forms of maltreatment, and (b) to explore whether self-compassion is indirectly associated with this relationship.

**Participants:**

A total of 143 adults aged 18–46 years (M = 23.26, SD = 2.90; 79.0% female) completed self-report online measures assessing childhood maltreatment, self-compassion, and social anxiety symptoms.

**Results:**

Hierarchical multiple regression was used to examine the relative contribution of emotional abuse to social anxiety after controlling for other maltreatment types. Indirect effects were tested using PROCESS Model 4. Emotional abuse emerged as the strongest predictor of social anxiety symptoms, accounting for unique variance beyond other forms of childhood maltreatment (β = .32, p < .001). Lower self-compassion was statistically associated with both greater emotional abuse exposure and higher social anxiety, yielding a significant indirect effect (indirect effect = .12, SE = .05, 95% CI [.04, .25]).

**Conclusions:**

Findings highlight the salience of childhood emotional abuse potentially reflecting chronic interpersonal invalidation in relation to adult social anxiety. Results suggest that self-compassion may represent a clinically relevant target for interventions aimed at individuals with histories of emotional abuse.

## Introduction

Child maltreatment refers to the mistreatment and neglect of children under the age of 18, encompassing both actual or potential threats of harm by parents or other caregivers (World Health Organization [WHO], 2006). It is widely recognized as a global public health concern, contributing to substantial societal and economic burden worldwide (WHO, 2022).

The WHO (2006) classifies child maltreatment into five subtypes: emotional abuse, emotional neglect, physical abuse, physical neglect, and sexual abuse. Emotional abuse involves caregiver behaviors that undermine a child’s emotional well-being or sense of self-worth, such as verbal hostility, belittling, rejection, and humiliation (Hart & Brassard, 1991). Emotional neglect refers to persistent failures to meet a child’s emotional needs, including emotional unavailability or exposure to domestic violence (Stoltenborgh et al., 2013). Physical abuse entails the intentional use of physical force that threatens or harms a child’s health or development (e.g., hitting, shaking, burning; Dubowitz & Bennett, 2007), whereas physical neglect involves failures to provide for basic physical needs such as education and medical care (Stoltenborgh et al., 2013). Sexual abuse includes coerced or forced sexual activity involving a child, such as penetration, fondling, or exposure to sexually explicit acts (Centers for Disease Control and Prevention, 2019). These forms of maltreatment are highly prevalent globally, with meta-analytic estimates indicating rates of 36.3% for emotional abuse, 18.5% for emotional neglect, 22.6% for physical abuse, 16.3% for physical neglect, and 12.7% for sexual abuse (Stoltenborgh et al., 2015).

Extensive research demonstrates that child maltreatment is associated with both immediate and long-term psychological consequences, often resulting in enduring trauma across the lifespan (Cay et al., 2022; Russotti et al., 2021). Individuals with histories of maltreatment are at heightened risk for externalizing difficulties (e.g., aggression, suicidality; Angelakis et al., 2020) as well as internalizing problems, including depression and anxiety (Gardner et al., 2019; Humphreys et al., 2020). Notably, emotional abuse has been found to show particularly strong associations with internalizing outcomes compared to other maltreatment subtypes, including social anxiety symptoms (Lochner et al., 2010; Nanda et al., 2016). However, findings regarding the extent to which emotional abuse uniquely contributes to social anxiety have been inconsistent, particularly when co-occurring forms of maltreatment are taken into account.

Importantly, emotional abuse rarely occurs in isolation and often overlaps with other adverse caregiving experiences, such as emotional neglect and attachment-related disruptions. As such, emotional abuse may function as an indicator of chronic interpersonal invalidation or relational trauma rather than a fully separable exposure. While this conceptual overlap complicates efforts to isolate its effects, examining the relative contribution of emotional abuse while statistically accounting for co-occurring maltreatment remains important for identifying particularly salient relational risk factors for social anxiety. Furthermore, considerable heterogeneity exists in outcomes following emotional abuse, as not all individuals exposed to emotional abuse develop clinically significant social anxiety symptoms (Iwaniec et al., 2006). This variability underscores the need to identify psychological processes that may help explain differential vulnerability.

One potential mechanism linking emotional abuse to social anxiety is self-compassion, defined as treating oneself with kindness, recognizing shared human imperfection, and maintaining balanced awareness of distressing experiences (Neff 2003b). Exposure to emotionally invalidating environments may undermine the development of self-compassion, which in turn could be associated with heightened fear of negative evaluation and social threat sensitivity (Wong et al., 2022). Although prior studies have reported bivariate associations among emotional abuse, self-compassion, and social anxiety (Humphreys et al., 2020; Nanda et al., 2016; Werner et al., 2012), few have examined self-compassion as a statistical intermediary in this relationship while accounting for the co-occurrence of other forms of childhood maltreatment. Given that maltreatment subtypes frequently co-occur (Kinard, 1994; van Veen et al., 2013), failure to control for these overlaps may obscure the relative salience of emotional abuse.

### Emotional Abuse and Social Anxiety

Social anxiety is characterized by a persistent fear of behaving in a manner that may result in embarrassment or humiliation when interacting with unfamiliar individuals or when under potential scrutiny by others (American Psychiatric Association, 2013). Individuals with social anxiety commonly fear becoming the focus of attention and being negatively evaluated. Behaviorally, they tend to avoid social situations or endure them with intense fear and anxiety (Myllyneva et al., 2015; Stein & Stein, 2008). Cognitively, social anxiety is associated with heightened self-focused attention, whereby individuals closely monitor their internal states, thoughts, and bodily sensations in an effort to prevent negative evaluation (Spurr & Stopa, 2002). Over time, these patterns may contribute to strained interpersonal interactions (Davila & Beck, 2002), difficulties forming and maintaining relationships (de Bérail et al., 2019; Tillfors et al., 2012), and elevated risk for broader psychological difficulties (Buckner et al., 2017; Epkins & Heckler, 2011). Accordingly, identifying developmental risk factors for social anxiety remains an important research priority.

Both theoretical models and empirical evidence suggest that childhood emotional abuse represents a particularly salient interpersonal risk factor for social anxiety. Ollendick and Benoit’s (2012) parent–child interaction model posits that caregiving environments characterized by criticism, rejection, and threat contribute to social anxiety by shaping maladaptive beliefs about self and others. Emotional abuse, which directly communicates devaluation and conditional acceptance, aligns closely with these mechanisms and may heighten sensitivity to rejection and fear of negative evaluation.

Consistent with this framework, several studies report stronger associations between emotional abuse and social anxiety compared to other maltreatment subtypes. For example, Gibb et al. (2007) found emotional abuse to be more strongly related to social anxiety than physical or sexual abuse, and a recent meta-analysis by Liu et al. (2023) similarly identified emotional maltreatment as the most robust correlate. Some studies further indicate that emotional abuse accounts for unique variance in social anxiety when other maltreatment types are controlled (e.g., Nanda et al., 2016). However, findings have not been entirely consistent, with some longitudinal studies failing to detect prospective effects (Calvete, 2014), suggesting that developmental timing, measurement approaches, or intervening psychological processes may moderate this association.

Given the frequent co-occurrence of maltreatment subtypes and mixed findings in the literature, re-examining the relative contribution of emotional abuse to adult social anxiety while accounting for other forms of maltreatment remains warranted.

### Self-compassion as a Mediator

Not all individuals exposed to emotional abuse develop elevated social anxiety, suggesting the presence of protective or buffering processes (Lochner et al., 2010). Identifying such mechanisms is critical for advancing prevention and intervention efforts. To date, however, relatively few studies have examined psychological processes that may account for variability in social anxiety following emotional abuse. Existing evidence points to factors such as shame-proneness, self-criticism, and self-esteem as potential mediators (Shahar et al., 2015; Chen & Qin, 2020), but this literature remains limited.

One construct that may be particularly relevant is self-compassion, defined as responding to personal suffering with kindness, recognition of shared humanity, and balanced awareness of distress (Neff 2003a, b). Self-compassion encompasses self-kindness, common humanity, and mindfulness, and may counteract excessive self-criticism and fear of negative evaluation—core features of social anxiety. Accordingly, individuals who adopt a compassionate stance toward perceived social shortcomings may experience reduced anxiety in evaluative contexts (Blackie & Kocovski, 2018).

Empirical studies consistently demonstrate inverse associations between self-compassion and social anxiety across age groups and populations. Lower self-compassion has been linked to higher social anxiety among adolescents (Gill et al., 2018), adults with social anxiety disorder (Werner et al., 2012), and community and student samples (Bates et al., 2021). Although the directionality of these associations cannot be inferred from cross-sectional data, the findings suggest that self-compassion is a psychologically meaningful correlate of social anxiety.

Converging evidence also indicates that emotional abuse is associated with reduced self-compassion. Emotionally abusive caregiving environments characterized by rejection and invalidation may undermine the development of a compassionate self-relation, fostering shame and chronic self-criticism (Miron et al., 2014, 2016). Consistent with this view, multiple studies report negative associations between emotional abuse and self-compassion (Kwok et al., 2022; Wu et al., 2018), with emotional abuse often demonstrating stronger associations than physical or sexual maltreatment (Miron et al., 2016; Zhang et al., 2023). These findings suggest that self-compassion may represent a plausible psychological process linking emotional abuse and social anxiety.

### The Current Study

The present study examined associations among childhood emotional abuse, self-compassion, and social anxiety symptoms in a general adult sample. Given mixed findings regarding whether emotional abuse predicts social anxiety when other forms of childhood maltreatment are considered, the first objective was to re-evaluate the relationship between emotional abuse and social anxiety while statistically accounting for co-occurring maltreatment experiences. Rather than treating emotional abuse as categorically distinct, this approach enabled assessment of its *relative salience* compared to other maltreatment subtypes.

**Fig. 1:**
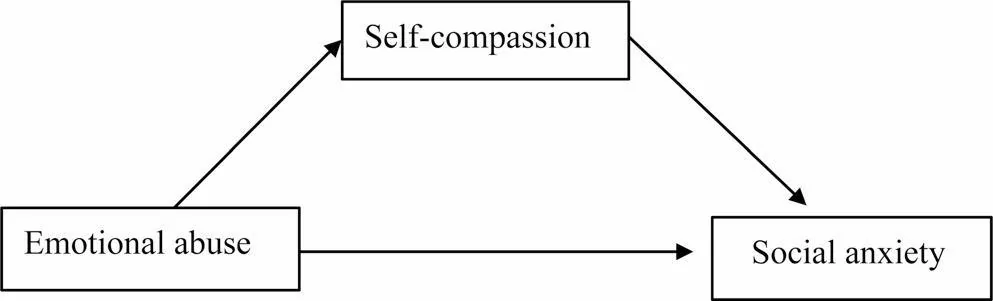
Hypothesized mediation model

The second objective was to examine whether self-compassion is statistically associated with the link between emotional abuse and social anxiety. Drawing on theoretical models and empirical evidence suggesting that emotionally abusive experiences may undermine self-compassion which in turn is associated with heightened social anxiety, the study tested an indirect (mediation) model (see Fig. 1).

Age and gender were included as covariates, given established associations with both self-compassion and social anxiety (Asher et al., 2017; Bluth et al., 2017; Caballo et al., 2008). In addition, other forms of childhood maltreatment were included as covariates to account for their high co-occurrence and to reduce confounding, allowing for a more precise estimation of variance uniquely associated with emotional abuse (Berzenski & Yates, 2011; Kinard, 1994; van Veen et al., 2013).

Based on the existing literature, the following hypotheses were tested:

- Hypothesis 1. Childhood emotional abuse will exhibit a stronger association with social anxiety symptoms than other forms of childhood maltreatment when all subtypes are considered simultaneously.
- Hypothesis 2. Self-compassion will be indirectly associated with the relationship between childhood emotional abuse and social anxiety symptoms, such that higher levels of emotional abuse will be associated with lower self-compassion, which in turn will be associated with greater social anxiety.

## Methods

### Design

A cross-sectional design was used to examine associations among childhood emotional abuse, self-compassion, and social anxiety symptoms. Hierarchical multiple regression analyses tested the unique contribution of emotional abuse to social anxiety while controlling for age, gender, and other forms of childhood maltreatment (emotional neglect, physical abuse, physical neglect, and sexual abuse). To examine indirect associations, a mediation model was specified with emotional abuse as the predictor, self-compassion as the mediator, and social anxiety as the outcome, with age, gender, and remaining maltreatment subtypes included as covariates.

### Participants

Participants were recruited via social media platforms (Facebook and Weibo) and the University online participant pool. Inclusion criteria required participants to be aged 18 years or older and proficient in English. Of the 151 individuals who consented, eight were excluded due to more than 50% missing data, yielding a final sample of 143 adults (age range = 18–46 years; *M* = 23.26, *SD* = 2.90).

The sample comprised 113 females (79.0%), 27 males (18.9%), and three non-binary individuals (2.1%). Most participants identified as Asian (86%), followed by White (9.1%), Hispanic (2.1%), Multiracial (1.4%), and Black (1.4%). Educational attainment was high school (8.4%), undergraduate (46.2%), graduate (44.1%), or undisclosed (1.4%).

An a priori power analysis conducted using G*Power 3.1 (Faul et al., 2009) indicated that a minimum sample of 138 participants was required to detect a medium effect size (*f*² = 0.15) with seven predictors at 95% power (α = 0.05). The final sample exceeded this threshold.

### Measures

#### Childhood Maltreatment

Childhood maltreatment was assessed using the Childhood Trauma Questionnaire (CTQ; Bernstein et al., 1994, 2003), a 28-item self-report measure assessing emotional abuse, emotional neglect, physical abuse, physical neglect, and sexual abuse. Items are rated on a 5-point scale (1 = *never true* to 5 = *very often true*), with higher scores indicating greater maltreatment exposure. Validity items assessing minimization/denial were excluded. Internal consistency in the present sample was excellent for the total scale (α = 0.93) and acceptable to high across subscales (αs = 0.71–0.92).

#### Self-compassion

Self-compassion was measured using the 12-item Self-Compassion Scale–Short Form (SCS-SF; Raes et al., 2011). Items are rated on a 5-point scale (1 = *almost never* to 5 = *almost always*), with higher scores reflecting greater self-compassion. Consistent with prior research, analyses focused on the total score. Internal consistency was acceptable (α = 0.81).

#### Social Anxiety

Social anxiety symptoms were assessed using the self-report version of the Liebowitz Social Anxiety Scale (LSAS-SR; Fresco et al., 2001). The scale comprises 24 items assessing fear and avoidance of social situations. Fear and avoidance are rated on separate 4-point scales, with higher scores indicating greater symptom severity. Internal consistency was excellent in the present sample (α = 0.96).

### Procedure

Data were collected via Qualtrics. After providing informed consent, participants completed a demographic questionnaire followed by the self-compassion, childhood maltreatment, and social anxiety measures. Upon completion, participants received a debriefing outlining the study aims, data confidentiality, and their right to withdraw. Survey completion took approximately 20 min.

### Data Analysis

Analyses were conducted using IBM SPSS Statistics (Version 29) and the PROCESS macro (Version 4.2; Hayes, 2013). Preliminary analyses included descriptive statistics and Pearson correlations.

Hierarchical multiple regression was used to examine the unique association between emotional abuse and social anxiety after controlling for age, gender, and other maltreatment subtypes. Indirect associations were examined using PROCESS Model 4, with 5,000 bootstrap samples used to estimate bias-corrected 95% confidence intervals. Indirect effects were considered statistically significant when confidence intervals did not include zero. Consistent with the cross-sectional design, analyses were interpreted as assessing statistical indirect associations rather than causal mediation.

### Ethics

Ethical approval was obtained from the University Research Ethics Committee. All participants provided informed consent and were informed of their right to withdraw without penalty. Given the sensitive nature of the content, participants were advised that they could discontinue participation if distressed and were provided with support resources. All data were anonymized and securely stored in accordance with data protection guidelines.

## Results

### Preliminary Analysis

Preliminary analyses were conducted to assess whether the data met the assumptions required for multivariate analyses. The Durbin–Watson statistic was 1.86, indicating that the assumption of independence of errors was satisfied. Inspection of residual scatterplots suggested that the assumption of homoscedasticity was met, and normal probability–probability (P–P) plots indicated that residuals were approximately normally distributed. Multicollinearity was not evident, as variance inflation factor (VIF) values were below 10 and tolerance values exceeded 0.25 for all predictors. Pearson correlation analyses (see Table 1) supported the assumption of linearity, and no excessively high intercorrelations (e.g., *r* > .90) were observed, indicating that the variables were suitable for further analyses (Preacher & Hayes, 2008).

**Table 1:** Means, standard deviations and correlations among childhood maltreatment subtypes, self-compassion and social anxiety

	1	2	3	4	5	6	7
1. Emotional Abuse							
2. Emotional Neglect	0.61^**^						
3. Physical Abuse	0.66^**^	0.29^**^					
4. Physical Neglect	0.63^**^	0.54^**^	0.67^**^				
5. Sexual Abuse	0.58^**^	0.23^**^	0.70^**^	0.60^**^			
6. Self-compassion	− 0.32^**^	− 0.23^**^	− 0.08	− 0.14	− 0.07		
7. Social Anxiety	0.32^**^	0.12	0.21^*^	0.17^**^	0.24^**^	− 0.33^**^	
*M*	9.32	10.83	7.78	8.52	6.65	37.09	58.15
*SD*	4.43	4.01	3.74	3.60	3.42	7.03	26.82

*M* Mean, *SD* Standard deviation

** *p* < .01; * *p* < .05

Outlier analysis using Mahalanobis distance identified two multivariate outliers exceeding the critical chi-square value at *p* < .001 (Tabachnick & Fidell, 2013). These cases were excluded from subsequent analyses, resulting in a final analytic sample of 143 participants.

### Descriptive Statistics and Correlations

Means, standard deviations, and Pearson correlation coefficients for all study variables are presented in Table 1. Among the child maltreatment subtypes, emotional abuse demonstrated statistically significant associations with both self-compassion and social anxiety symptoms. Specifically, higher levels of emotional abuse were associated with lower self-compassion, r(139) = − 0.32, *p* < .01, and higher levels of social anxiety, r(139) = 0.32, *p* < .01.

Emotional neglect was also negatively associated with self-compassion, r(139) = − 0.23, *p* < .01. In addition, social anxiety showed positive associations with physical neglect, r(139) = 0.17, *p* < .05, physical abuse, r(139) = 0.21, *p* < .05, and sexual abuse, r(139) = 0.24, *p* < .01. Finally, self-compassion was negatively associated with social anxiety symptoms, r(139) = − 0.33, *p* < .01.

Overall, the correlational pattern suggests that while multiple forms of childhood maltreatment were associated with social anxiety at the bivariate level, emotional abuse was uniquely associated with both lower self-compassion and higher social anxiety, supporting its relevance for subsequent regression and indirect association analyses.

### Regression Analysis

A hierarchical multiple regression was conducted to examine which types of child maltreatment can predict social anxiety. Demographic variables, including age and gender (0 = male,1 = female, 2 = non-binary), were entered in the first step. Subsequently, emotional abuse, emotional neglect, physical abuse, physical neglect and sexual abuse were entered in that order for the second through sixth steps. The sequencing of these steps was determined based on the prior research. Hence, a total of six models were examined, with each one being significant (see Table 2).

**Table 2:** Hierarchical multiple regression analysis for childhood maltreatment subtypes predicting social anxiety

	Model 1		Model 2		Model 3		Model 4		Model 5		Model 6	
	B (SE B)	β	B (SE B)	β	B (SE B)	β	B (SE B)	β	B (SE B)	β	B (SE B)	β
1	-2.696 (1.03)	-0.22**	-2.54 (0.98)	-0.21*	-2.51 (0.98)	-0.20*	-2.52 (0.98)	-0.20*	-2.52(0.99)	-0.20*	-2.47 (0.99)	-0.20*
2	-1.38 (5.18)	-0.02	-1.61 (4.92)	-0.03	-1.19 (4.93)	-0.02	-1.34 (4.97)	-0.02	-1.36 (5.00)	-0.02	-1.66 (5.03)	-0.03
3			1.91 (0.48)	0.32***	2.32 (0.60)	0.38***	2.49 (0.78)	0.41**	2.49 (0.79)	0.41**	2.38 (0.81)	0.39**
4					-0.75 (0.67)	-1.11	-0.79 (0.68)	-1.12	-0.78 (0.74)	0.12	-0.68 (0.75)	0.10
5							-0.26 (0.77)	-0.04	-0.24(0.89)	0.03	-0.46 (0.96)	0.06
6									-0.05 (0.91)	0.01	-0.21 (0.95)	0.03
7											-0.60 (0.94)	0.08
Adjusted R^2^	0.03		0.13		0.13		0.12		0.12		0.11	
△F	3.48		15.88***		1.28		0.12		0.00		0.3	
F	3.48*		7.87***		6.23***		4.98***		4.12***		4.57***	

*** p < .001 ** p < .01; * *p* < .05

(1) Age, (2) Gender, (3) Emotional Abuse, (4) Emotional Neglect, (5) Physical Abuse, (6) Physical Neglect, (7) Sexual Abuse

The results showed that emotional abuse was a strong predictor for social anxiety (*β* = 0.32, *p* < .001), explaining an additional 9.9% variance beyond demographic variables in social anxiety. Notably, emotional abuse remained a significant predictor even when other variables were included in the model. In contrast, none of the other types of child maltreatment made a significant contribution to predicting social anxiety. Specifically, emotional neglect (*β* = − 0.11, *p* > .05) only accounted for an additional 0.8% of variance beyond Model 2. Physical abuse (*β* = − 0.04, *p* > .05) accounted for an additional 0.1% of variance beyond Model 3. Physical neglect (*β* = − 0.01, *p* > .05) accounted for an additional 0.0% of variance beyond Model 4. Sexual abuse (*β* = − 0.08, *p* > .05) accounted for an additional 0.3% of variance beyond Model 5.

Collectively, the hierarchical multiple regression analysis demonstrated that emotional abuse retained its significance as a predictor of social anxiety, even when considering all other types of childhood maltreatment. In contrast, none of the other types of childhood maltreatment played a role in predicting social anxiety.

### Mediation Analysis

A mediation analysis was conducted to examine whether self-compassion was indirectly associated with the relationship between childhood emotional abuse and social anxiety symptoms. Age, gender, physical abuse, sexual abuse, emotional neglect, and physical neglect were included as covariates. Consistent with the cross-sectional design, this analysis was intended to assess statistical indirect associations among variables rather than temporal or causal mediation.

Results indicated that the total association between emotional abuse and social anxiety symptoms was statistically significant, b = 2.38, t = 2.94, *p* < .01, 95% CI [0.78, 3.97]. When self-compassion and covariates were included in the model, the direct association between emotional abuse and social anxiety remained statistically significant, β = 0.27, t = 2.01, *p* < .05.

Emotional abuse was significantly and negatively associated with self-compassion, β = −0.47, t = − 3.37, *p* < .001. In turn, lower self-compassion was significantly associated with higher social anxiety symptoms, β = −0.26, t = − 3.13, *p* < .01. Bootstrapping analyses (5,000 samples) indicated a statistically significant indirect association between emotional abuse and social anxiety through self-compassion (indirect effect = 0.12, SE = 0.05, 95% CI [0.04, 0.25]), as the confidence interval did not include zero.

Overall, the model accounted for 15.8% of the variance in social anxiety symptoms. These findings support the presence of a statistically significant indirect association among emotional abuse, self-compassion, and social anxiety while acknowledging that alternative directional or third-variable explanations remain plausible.

## Discussion

The present study examined associations among childhood emotional abuse, self-compassion, and social anxiety in a general adult sample, while accounting for other forms of childhood maltreatment. Specifically, the study aimed to test to what extent emotional abuse predicts social anxiety while accounting for other types of child maltreatment. Secondly, it aimed to explore whether self-compassion was statistically linked to this association. Overall, the findings indicate that emotional abuse showed a relatively robust association with social anxiety compared to other maltreatment subtypes, and that self-compassion was significantly associated with both emotional abuse and social anxiety, forming a statistically significant indirect association. Importantly, the observed effects were not only statistically significant but also of a magnitude suggesting meaningful psychological relevance, highlighting emotional abuse as a clinically important correlate of social anxiety symptoms.

### Emotional Abuse and Social Anxiety

Consistent with Hypothesis 1, emotional abuse was uniquely associated with social anxiety symptoms after accounting for demographic variables and other forms of childhood maltreatment. Although several maltreatment subtypes demonstrated bivariate associations with social anxiety, emotional abuse remained the most salient factor when considered simultaneously, suggesting that it may be particularly relevant to social anxiety symptomatology.

This pattern aligns with prior empirical work indicating that emotional abuse is more strongly linked to internalizing difficulties than physical or sexual abuse (Gibb et al., 2007; Liu et al., 2023; Nanda et al., 2016). The results are also broadly consistent with the parent–child interaction model of social anxiety (Ollendick & Benoit, 2012), which emphasizes the role of parental behaviors that convey rejection, criticism, or threat in shaping children’s self-referential beliefs and fear of negative evaluation. While the present study cannot speak to developmental sequencing, the observed associations lend further support to the theoretical relevance of emotionally abusive caregiving experiences in relation to social anxiety.

One possible explanation for the differential associations across maltreatment subtypes lies in the cognitive sequelae associated with emotional abuse. Cognitive models of social anxiety highlight the central role of negative self-beliefs, heightened self-consciousness, and fear of negative evaluation (Clark & Wells, 1995). Emotional abuse, characterized by persistent criticism, humiliation, or rejection, may be particularly likely to foster such maladaptive cognitive styles compared to more episodic or less self-referential forms of maltreatment. Empirical evidence suggests that emotional abuse is more strongly associated with negative cognitive styles and reduced self-esteem than physical or sexual abuse (Gibb, 2002; van Harmelen et al., 2010; Zhang et al., 2023). In contrast, emotional neglect, which is more passive in nature, may exert weaker effects on explicit self-evaluative cognitions because it does not necessarily involve direct negative feedback about the self (Golden et al., 2003). These distinctions may help explain why emotional abuse demonstrated a stronger association with social anxiety in the present study.

### Self-Compassion as an Indirectly Associated Mechanism

Consistent with Hypothesis 2, the mediation analyses indicated that self-compassion was statistically associated with the link between emotional abuse and social anxiety, even after controlling for age, gender, and other forms of childhood maltreatment. Specifically, higher levels of emotional abuse were associated with lower self-compassion, which in turn was associated with greater social anxiety symptoms. These findings suggest that self-compassion may represent an important indirectly associated mechanism linking emotional abuse and social anxiety. However, given the cross-sectional nature of the data, this mediation should be interpreted as reflecting an indirect association rather than a temporal or causal process.

The present findings are consistent with prior research demonstrating that self-compassion is associated with reduced psychological distress among individuals with a history of emotional abuse (Huang & Hou, 2023; Ross et al., 2019). For example, higher self-compassion has been shown to attenuate associations between emotional abuse and outcomes such as suicidal ideation (Huang & Hou, 2023) and depressive symptoms (Ross et al., 2019). Extending this literature, the current study provides preliminary evidence that self-compassion is also statistically linked to social anxiety in the context of emotional abuse, thereby broadening the range of trauma-related outcomes with which self-compassion appears to be associated.

Beyond the overall indirect association, it is informative to consider the individual pathways within the proposed model. First, emotional abuse was negatively associated with self-compassion, a finding that converges with previous research indicating that emotionally abusive experiences may undermine individuals’ capacity for self-kindness and self-acceptance (Kwok et al., 2022; Miron et al., 2014; Wu et al., 2018). From an attachment theory perspective, self-compassion is thought to emerge partly through early caregiving experiences, whereby children internalize caregivers’ responses to distress and gradually develop self-soothing capacities (Gilbert, 2005, 2009). Supportive and validating caregiving may facilitate the development of compassionate self-responding, whereas emotionally abusive caregiving characterized by criticism, rejection, or hostility that may foster internalized self-criticism and reduced self-compassion (Neff & McGehee, 2010). Consequently, individuals with histories of emotional abuse may be less inclined to respond to personal difficulties with kindness and understanding.

Second, self-compassion was negatively associated with social anxiety, consistent with a growing body of research suggesting that individuals higher in self-compassion tend to report lower levels of social evaluative fear and avoidance (Bates et al., 2021; Gill et al., 2018; Werner et al., 2012). Theoretically, self-compassion may be associated with social anxiety through several processes. Individuals with greater self-compassion may allocate fewer cognitive resources to concerns about negative evaluation and engage less in self-focused attention during social interactions, allowing for a more balanced and flexible appraisal of social situations (Gill et al., 2018). In addition, recognizing social mistakes or embarrassment as part of shared human experience may reduce avoidance-based coping strategies and lessen the perceived threat of social evaluation (Leary et al., 2007).

Nevertheless, alternative explanations for the observed mediation pattern should be considered. It is plausible that elevated social anxiety may erode self-compassion over time, or that both constructs are influenced by third variables such as shame-proneness, attachment insecurity, or self-criticism. Future longitudinal and experimental research will be essential to clarify the directionality and specificity of these associations.

### Clinical Implications

Although the present sample was non-clinical, the findings may have potential relevance for trauma-informed clinical practice. Prior research suggests that different subtypes of childhood maltreatment are associated with distinct psychological outcomes (Kim & Cicchetti, 2010). Consistent with this literature, the current study found that emotional abuse was uniquely associated with social anxiety when other maltreatment subtypes were considered simultaneously. This finding underscores the clinical importance of assessing emotional abuse histories in individuals presenting with social anxiety symptoms, particularly given that emotional abuse may be less readily identified than other forms of maltreatment.

Importantly, the findings should not be interpreted as evidence of treatment efficacy. Rather, they suggest that self-compassion may represent a clinically relevant process associated with social anxiety among individuals with histories of emotional abuse. Given that childhood maltreatment itself is not modifiable, identifying malleable psychological processes linked to trauma-related distress remains an important clinical objective. In this context, social anxiety symptoms may constitute a meaningful intervention target for individuals with emotional abuse histories.

A range of compassion-based interventions—including Mindfulness-Based Stress Reduction (MBSR; Shapiro et al., 2005), Compassionate Mind Training (CMT; Gilbert & Irons, 2005), Mindful Self-Compassion (MSC; Neff & Germer, 2013), Cognitive-Based Compassion Training (CBCT; Reddy et al., 2013), and Compassion-Focused Therapy (CFT; Gilbert, 2009)—have demonstrated efficacy in enhancing self-compassion and reducing psychological distress. However, the present findings do not suggest that increasing self-compassion alone is sufficient to alleviate trauma-related social anxiety. Rather, compassion-based approaches may be most appropriately conceptualized as adjunctive components within broader, trauma-informed treatment frameworks that also address attachment insecurity, shame, self-criticism, and interpersonal avoidance.

### Limitations and Future Directions

Several limitations of the present study should be acknowledged. First, childhood maltreatment was assessed using retrospective self-report measures, which are vulnerable to recall bias and social desirability effects (Brewin et al., 1993; Podsakoff et al., 2003). Although self-report instruments such as the CTQ are widely used and psychometrically robust, future research may benefit from incorporating multimethod assessments, including structured clinical interviews or corroborative reports, where feasible.

Second, the cross-sectional design precludes conclusions regarding temporal ordering or causality among emotional abuse, self-compassion, and social anxiety. While the mediation analysis identified a statistically significant indirect association, alternative models are equally plausible. Longitudinal designs and experimental studies that manipulate self-compassion are needed to more definitively evaluate developmental pathways and mechanisms.

Third, the study focused on a single mediator. Future research could expand this model by examining additional psychological processes implicated in trauma-related social anxiety, such as shame, emotion regulation, attachment representations, or perceived social support. Examining multiple mediators simultaneously may help clarify whether self-compassion represents a distinct mechanism or overlaps with related constructs such as self-criticism.

Finally, the generalizability of the findings is limited by sample characteristics. The sample was predominantly female and largely Asian, which warrants caution in extending conclusions to other populations. Cultural norms regarding parenting practices, emotional expression, and self-compassion may shape how emotional abuse is experienced and internalized. For example, self-compassion may function differently in collectivist cultural contexts, where self-criticism and interpersonal sensitivity may be more normative. Future research should aim to include more gender-balanced and culturally diverse samples to clarify the cultural specificity of these associations.

## Conclusion

The present study examined the associations among emotional abuse, self-compassion, and social anxiety in a general adult sample. Emotional abuse emerged as a particularly salient form of childhood maltreatment associated with social anxiety when accounting for other maltreatment subtypes. In addition, self-compassion was indirectly associated with this relationship, suggesting that it may represent a relevant psychological process linking emotional abuse and social anxiety.

These findings contribute to the trauma literature by highlighting the potential importance of emotional abuse and self-compassion in understanding social anxiety symptoms. However, conclusions regarding causality and intervention efficacy are necessarily limited by the cross-sectional design. Future longitudinal and experimental research is needed to clarify the developmental role of self-compassion and its place within trauma-informed interventions for social anxiety.

## Data Availability

The datasets generated and/or analysed during the current study are not publicly available due to ethical and confidentiality considerations but are available from the corresponding author on reasonable request.
